# Did the movie *Finding Dory* increase demand for blue tang fish?

**DOI:** 10.1007/s13280-019-01233-7

**Published:** 2019-08-14

**Authors:** Diogo Veríssimo, Sean Anderson, Michael Tlusty

**Affiliations:** 1 Oxford Martin School, University of Oxford, Oxford, UK; 2 San Diego Zoo Institute for Conservation Research, Escondido, CA USA; 3 Pacific Biological Station, Fisheries and Oceans Canada, 3190 Hammond Bay Rd, Nanaimo, BC V6T 6N7 Canada; 4 School for the Environment, University of Massachusetts Boston, Boston, MA 02125 USA; 5 Department of Zoology, University of Oxford, 11a Mansfield Road, Oxford, OX1 3SZ UK

**Keywords:** Behaviour change, Consumer, Demand, Impact evaluation, Ornamental fish, Wildlife trade

## Abstract

Representations of wildlife in television and films have long been hypothesized to shape human-wildlife interactions. A recent example is Pixar’s film *Finding Dory,* which featured a blue tang fish (*Paracanthurus hepatus*) as the main character and was widely reported in the popular press to have increased the number of such fish in the pet trade. We use Bayesian posterior predictive counterfactual models to evaluate the movie’s effect on three metrics of societal behaviour. Although there was an increase in global online searches for the blue tang 2–3 weeks after the movie, we find no substantial evidence for an increase in imports of blue tang fish into the US, or in number of visitors to US aquaria compared to counterfactual expectations. It is vital that an evidence-based discourse is used when communicating potential impacts of popular culture on human-wildlife relationships to avoid loss of credibility and misdirection of conservation resources.

**Electronic supplementary material:**

The online version of this article (10.1007/s13280-019-01233-7) contains supplementary material, which is available to authorized users.

## Introduction

For decades, movies and television series featuring animals have been perceived to influence the attitudes and behaviours of their audience toward nature (Hastings 1996; Yong et al. 2011). This influence has been portrayed as both positive and negative, since movies could both heighten concern for the natural world, as well as become a substitute for the natural world, thereby reducing the value of maintaining actual biodiversity (Silk et al. 2018). The weight of TV series and movies, when it comes to mediating our relationship with nature, is likely to increase over time as the flow of people towards urban areas decreases the opportunities for direct contact with nature (Pergams and Zaradic 2008). This is particularly the case for those TV series and movies that aim to entertain, as they are more likely to reach larger tracts of the population than, for example, nature documentaries, as these are likely to be viewed mainly by those with a specific interest in nature (Silk et al. 2018).

One of the first movies to reportedly impact societal perceptions of wildlife was Disney’s *Bambi*, released in 1942, which has long been seen as influential in decreasing the popularity of hunting in the United States (Hastings 1996). Other examples are characters such as Lassie and movies such as *101 Dalmatians*, which have since then been reportedly responsible for influencing preferences for domestic dog breeds in the USA (Ghirlanda et al. 2014). More recent examples of movies widely linked with the pet trade are the *Teenage Mutant Ninja Turtle* and *Harry Potter* franchises, which have been linked with the trade in terrapins and owls respectively, and Disney’s 2016 release *Zootopia*, which has been hypothesized to have generated demand for Fennec foxes *Vulpes zerda* (Yong et al. 2011; Veríssimo and Wan 2016; Megias et al. 2017). These associations have become so commonplace in the popular media that there are even instances where promotional offers for pet purchasing are linked to the launch of movies featuring animals (PETA 2011). Yet, there is a dearth of evidence to substantiate any causal impact of these media phenomena on audience behaviour and consequently on biodiversity (Megias et al. 2017; Silk et al. 2018).

### The “Nemo effect” narrative

The most global and often repeated example of a movie being perceived to impact consumer behaviour is perhaps Pixar’s 2003 production *Finding Nemo*. In the months following its release, popular media outlets worldwide reported an alleged steep rise in demand for the species of the movie’s protagonist, the clownfish (*Amphiprion ocellaris*/*percula)* for the pet trade. These outlets included major global news providers, such as the British Broadcasting Corporation (BBC), Cable News Network (CNN), USA Today, Public Broadcasting Service (PBS) (BBC 2003; CNN 2003; USA Today 2003; PBS 2004). The global media reach of this story amplified the narrative and turned it into conventional wisdom, ultimately coining the term *Nemo effect* to describe the impact of the movie on the fisheries of the film’s protagonist: the clownfish Nemo (Militz and Foale 2017). Despite it relying on anecdotal information, the narrative around the *Nemo effect* soon seeped into the academic literature, initially through articles that referenced popular media sources (Yong et al. 2011; Rhyne et al. 2012), but which were afterwards used as primary references in subsequent papers (e.g. Wunder and Sheil 2013; Bush et al. 2014).

With the *Nemo effect* solidified, there was another round of global media coverage in the run-up to the release of the film *Finding Dory, Finding Nemo*’s sequel. This involved media outlets including the Washington Post, The Times of India, Huffington Post and the Australian Broadcasting Corporation (Andrews 2016; Australian Broadcasting Corporation 2016; Huffington Post 2016; Times of India 2016). These articles expanded the *Nemo effect* narrative to a new species, questioning whether the movie’s sequel would have the same impact on the fisheries of the blue tang (*Paracanthurus hepatus*), the species of the film’s protagonist Dory. The blue tang is a marine ornamental fish, distributed across much of the Indian and Pacific Oceans and currently considered as Least Concern by the International Union for the Conservation of Nature (IUCN) (McIlwain 2012).

The increased sales of *P. hepatus* could constitute a threat to wild populations, since in contrast with clownfish, a species for which aquaculture is a common source of animals for the trade, there are no alternatives to wild caught *P. hepatus* specimens (Militz and Foale 2017). This new flurry of media attention was amplified by conservationists and several social influencers. Examples of this included the creation of the Australian non-governmental organization Saving Nemo (www.savingnemo.org), and the American talk show host Ellen DeGeneres issuing a public appeal to consumers not to buy *P. hepatus* (Polowy 2015; Dengate 2016). Despite the strength of this narrative in the popular media, the only peer-reviewed analysis of clownfish sales to date did not support the existence of a *Nemo effect* (Militz and Foale 2017). Yet, this analysis was limited by the lack of historical import data for clownfish and related species. This gap makes it difficult to establish a credible counterfactual (i.e. what would have happened if the movie had not been released) and derive robust conclusions about cause-effect relationships.

**Fig. 1:**
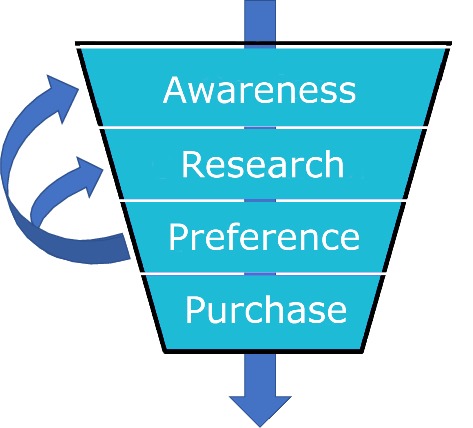
The Purchase funnel marketing model, describing the different stages that precede the purchasing of a product or service. Modified from Steve Simple CC BY 3.0

The aim of this paper is to use a counterfactual-based impact evaluation framework to measure the possible impact the movie *Finding Dory* had on the behaviour of consumers. We examine outcomes across the different stages that constitute a purchasing decision, as defined by the purchase funnel marketing model (Fig. 1) to be able to understand not only responses linked to purchases but also behaviours that precede it (Jansen and Schuster 2011). This model divides purchasing decisions into stages—it starts with awareness, moves into research, decision and finally purchase, while emphasizing how consumers can move forward and backward across the model (Jansen and Schuster 2011). The focus on a more recent movie release allows us to use richer data sets to test causality, in turn fulfilling the need recognized in the conservation literature of more evidence-based narratives when discussing the impact of entertainment media on our relationship with nature (Megias et al. 2017; Silk et al. 2018).

## Materials and Methods

### Counterfactual approach

Our approach was to build counterfactual regression models based on data from before the Dory movie release for different stages of the purchase process for *P. hepatus* (Dory’s species). We use these models to project our expectation of that variable of interest after the movie release and compare the observed level of interest to the counterfactual expectation of interest. This approach aligns conceptually with the synthetic control method, previously used in conservation science to investigate the impact of protected areas on deforestation (Sills et al. 2015). We built three counterfactual models. The first model focused on global Google search frequency, a type of indicator increasingly used in the biodiversity conservation literature as an indicator of societal interest in a species or topic (Nghiem et al. 2016). This information seeking behaviour fits into the research stage of the purchase funnel (Fig. 1), as it is an example of an action that may be undertaken before a decision on whether to make a purchase is made. The second model focused on aquarium fish imports in the US, a metric that reflects purchasing behaviour, the last stage of the purchase funnel model and the one that more closely relates to actual impacts on fish populations. Approximately 100 000 *P. hepatus* are imported yearly into the US from 25 countries, although the vast majority of these originate from Indonesia and the Philippines (www.aquariumtradedata.org). The species is the most traded in its family, accounting for about half of all imports into the USA of Acanthuridae fish. The species *Acanthurus japonicus* and *Acanthurus leucosternon* make up the majority of the remaining imports (www.aquariumtradedata.org). There are no restrictions to the trade in this fish species.

The third model focused on visitation patterns to US Aquaria, as we aimed to explore other potential behavioural outcomes of the “decision” stage of the purchase funnel model (Fig. 1), which could have led to outcomes other than purchases. With “*Finding Dory*” being a movie targeted at young audiences and *P. hepatus* being considered a species for advanced hobbyists, there is the potential for children or their parents to opt for an aquarium visit as a substitute for the time and resources needed to keep *P. hepatus*. In fact, past qualitative research into motivations of visitors to Aquaria in Canada, has found that some visitors mention movies, including *Finding Nemo,* as motivations for visiting (Briseno-Garzon et al. 2007).

We fit each model in a Bayesian framework to allow us to treat the predictions probabilistically and derive conclusions about the probability of effects of the movie release on purchase or likelihood of purchase. We fit all models with the probabilistic programming language Stan (Carpenter et al. 2017) using the packages brms (Bürkner 2017) and rstan (Stan Development 2018) for the statistical software R (R Core Team 2018). In all cases, we sampled from the posterior with 2000 iterations across each of four chains and discarded the first half as warm-up. We ensured consistency with chain convergence by inspecting trace plots, ensuring $\hat{R}$ (the potential scale reduction factor) was less than 1.05 for all parameters, and ensuring the minimum effective sample size *n*_eff_ was greater than 100 for all parameters (Gelman et al. 2014).

### Online searches

For this analysis, we first selected the species that would form the counterfactual. We selected species that were biologically close to *P. hepatus*, that shared key physical traits, such as size and broadly similar vivid colours (Fig. S1) and that were traded in the ornamental fish market. These included Tang fish species from the genera Acanthurus (*A. coeruleus*, *A. leucosternon*, *A. nigricans*, *A. sohal* and *A. japonicus*), and Zebrasoma (*Zebrasoma xanthurum*) both of which share the family Acanthuridae with *Paracanthurus.* On May 1, 2018 we retrieved relative global Google search frequency for each of the species scientific name, from January 1, 2014 to July 10, 2017 on a weekly basis using the R package gtrendsR (Massicotte and Eddelbuettel 2018) on January 21, 2018 (Fig. S2). We used the species scientific names as keywords to avoid overlaps in the common names of different species, for example with two species sharing the common name “blue tang”. We included the option “low_search_volume = TRUE”, which also includes searches from lower search volume regions of the world. We fit the models as beta regressions:

$$ \begin{aligned} y_{i} \sim {\text{Beta}}\left( {\mu_{i} ,\phi } \right) \hfill \\ {\text{logit}}\left( {\mu_{i} } \right) = {\mathbf{X}}_{i} {\varvec{\upbeta}}, \hfill \\ \end{aligned} $$

where

$$ {\text{logit}}\left( {\mu_{i} } \right) = { \log }\left( {\frac{{\mu_{i} }}{{1 - \mu_{i} }}} \right), $$

And *y*_*i*_ represents the observed proportion of global Google searches for *P. hepatus* in week *i*, **X**_*i*_ represents a vector of predictors with each element representing either the relative search frequency of a counterfactual species or the first-order interaction between them and **β** representing a vector of regression parameters including an intercept. We substituted 0.99 for a single week that had the maximum observed search frequency for *P. hepatus* (1.0) to avoid the complexity of a one-inflated beta-regression since the beta distribution cannot model 0 s or 1 s. We used the mean-precision parameterization of the beta distribution commonly used for regression purposes, which replaces the usual ${\text{Beta}}\left( {p,q} \right)$, with ${\text{Beta}}\left( {\mu ,\phi } \right)$ by setting $\mu = p/\left( {p + q} \right)$ and $\phi = p + q$ (Ferrari and Cribari-Neto 2004). We placed weakly informative priors of ${\text{Normal}}\left( {0, 2} \right)$ on the slope coefficients, ${\text{Normal}}\left( {0, 10} \right)$ on the intercept coefficient, and Half-Student-t(3, 0, 25) on the Beta precision parameter $\phi$.

### Imports

To reflect purchase patterns, we used monthly import data for 2015 and 2016, provided by Quality Marine, one of the US’ largest ornamental fish wholesalers (www.qualitymarine.com). We focused on the US as it was the largest market for the movie *Finding Dory* and was, therefore, the country where an impact was most likely. For this analysis, we used the same counterfactual species and overall approach detailed in the previous section (Fig. S3). We excluded *Z. xanthurum and A. sohal* due to the time series for these species being overwhelmingly dominated by zeros (Fig. S3). We modelled imports of *P. hepatus* with a negative binomial GLM (Generalized Linear Model) as:

$$\begin{array}{l}   y_{i} \sim {\text{NegativeBinomial}}\left( {\mu _{i} ,\phi } \right) \hfill \\   \log \left( {\mu _{i} } \right) = {\mathbf{X}}_{i} ,{\varvec{\upbeta}},  \hfill \\  \end{array}$$

Where *y*_*i*_ represents the observed number of imports of *P. hepatus* in month, *i*, **X**_i_ represents a vector of predictors with each element representing the number of imports for a counterfactual species in that month and **β** representing a vector of regression parameters including an intercept. We used the “NB2” parameterization of the negative binomial where the variance increases quadratically with linear increases in the mean $\left( {{\text{variance}} = \mu + \mu^{2} /\phi } \right)$, and that relationship is controlled by the dispersion parameter $\phi$ with smaller values of $\phi$ corresponding to more dispersed data (Hilbe 2011). We placed weakly informative priors of ${\text{Normal}}\left( {0, 2} \right)$ on the slope coefficients, ${\text{Normal}}\left( {0, 10} \right)$ on the intercept coefficient, and Half-Student-t(3, 0, 25) on the negative binomial dispersion parameter $\phi$.

### Aquarium visits

We used monthly aquarium attendance reported by 20 public and private institutions in the USA. These institutions varied considerably in size, with annual visitation varying between 10 000 and 2 400 000 visitors. Here we present an analysis of the summed visitor counts across all aquaria due to a data sharing agreement. We modelled the natural logarithm of the number of aquaria visits each month from January 1, 2006 to December 1, 2016 with an additive model as:

$$ \begin{aligned} { \log }\left( {y_{i} } \right)\sim {\text{Normal}}\left( {\mu_{i} ,\sigma^{2} } \right) \hfill \\ \mu_{i} = {\mathbf{M}}_{{\mathbf{i}}} {\varvec{\upbeta}}_{1} + s\left( {T_{i} } \right), \hfill \\ \end{aligned} $$

Where *y*_i_ represents 100,000 aquarium visits for point in time *i*, ${\mathbf{M}}_{{\mathbf{i}}}$ represents a factor predictor for month: either a vector of 0 s for the base month (January) or a vector of 0 s with a single 1 indicating the respective month, and $s\left( {T_{i} } \right)$ represents a smooth function over time. We represented time *T* as the decimal date (e.g. February 1st, 2014 = 2014.085) standardized by centring on its mean and scaling by two times its standard deviation to produce coefficients on an appropriate scale for the priors. The ${\varvec{\upbeta}}_{1}$ symbol represents a vector of coefficients and *σ* represents the residual standard deviation. We placed weakly informative priors of ${\text{Normal}}\left( {0, 2} \right)$ on the slope coefficients, ${\text{Normal}}\left( {0, 10} \right)$ on the intercept coefficient, and Half-Student-t(3, 0, 2) on *σ* and the standard deviation of the parameter describing the wiggliness of the spline smoother (Bürkner 2017).

## Results

### Google search frequency

**Fig. 2:**
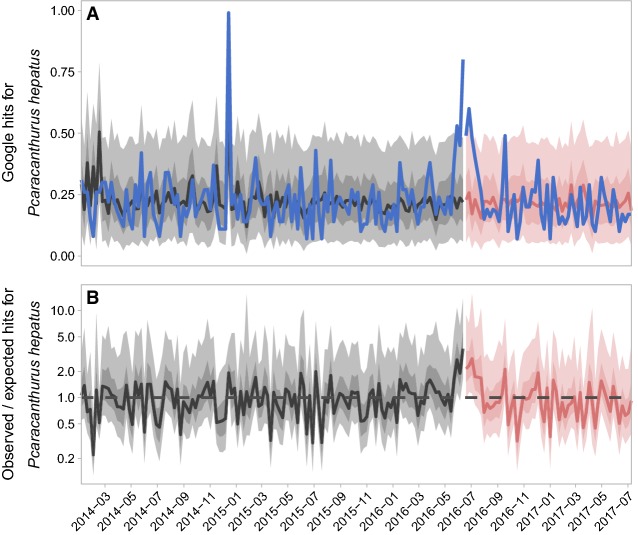
Google search frequency: **a** relative frequency of searches for *P. hepatus* (blue line) and counterfactual posterior predictive distribution based on similar species (shaded band). Black band represents the time period the counterfactual model was fit to and the red band represents the forecasted posterior predictive distribution after the release of the Dory movie. **b** Ratio of observed search frequency (blue line in **a**) to expected search frequency (shaded band in **a**) where values greater than 1 represent a higher than expected search frequency. Black/red lines and dark and light shaded regions represent median, 50%, and 90% credible intervals, respectively

The week after the movie *Finding Dory* was released, we found a search frequency 2.1 (90% credible interval [CI] 0.7–4.6) times higher for *P. hepatus* than predicted by the counterfactual model (Fig. 2, Fig. S4). This represents a 0.94 probability that there was a higher search frequency than expected in the week after the movie, conditional on our model and data. In the second and third weeks after the movie, there was a 0.98 and 0.97 probability of a higher search frequency than expected. However, the search frequency for *P. hepatus* returned to expected levels within approximately 2 months with only a 0.29 probability of higher than expected search frequency by week 7 after the movie was released. We also detected a peak in searches in late 2014, which could not be explained by variation in the counterfactual model.

### Imports

**Fig. 3:**
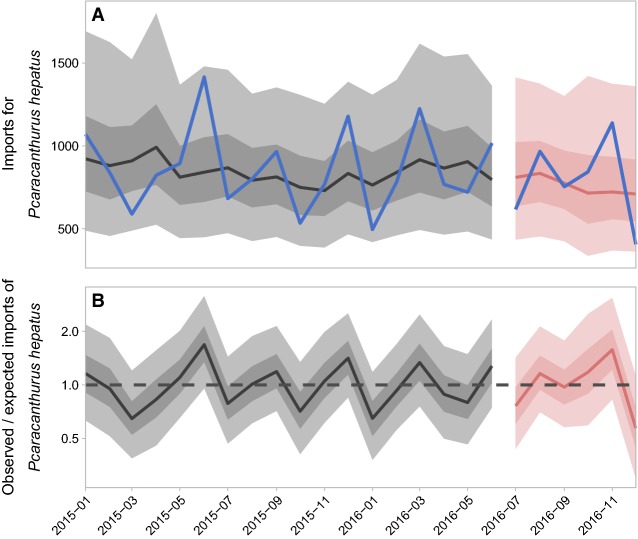
Imports: **a** number of imports of *P. hepatus* (blue line) and counterfactual posterior predictive distribution based on similar species (shaded band). Black band represents the time period the counterfactual model was fit to and the red band represents the forecasted posterior predictive distribution after the release of the Dory movie. **b** Ratio of observed imports (blue line in **a**) to expected imports frequency (shaded band in **a**) where values greater than 1 represent a higher than expected number of imports. Black/red lines and dark and light shaded regions represent median, 50%, and 90% credible intervals, respectively

In the months after the movie was released, the posterior prediction from the counterfactual model suggested a low probability of an effect on imports of *P. hepatus* (Fig. 3, Fig. S5). For example, in July 2016 (1 month after the release of *Finding Dory*), we found observed imports of *P. hepatus* were only 0.8 (90% CI 0.4–1.4) times the expected imports from the counterfactual model. This translates to only a 0.22 probability that the imports were greater than expected. The following month, there was still a low probability that the imports were greater than expected (0.68).

### Aquarium visits

**Fig. 4:**
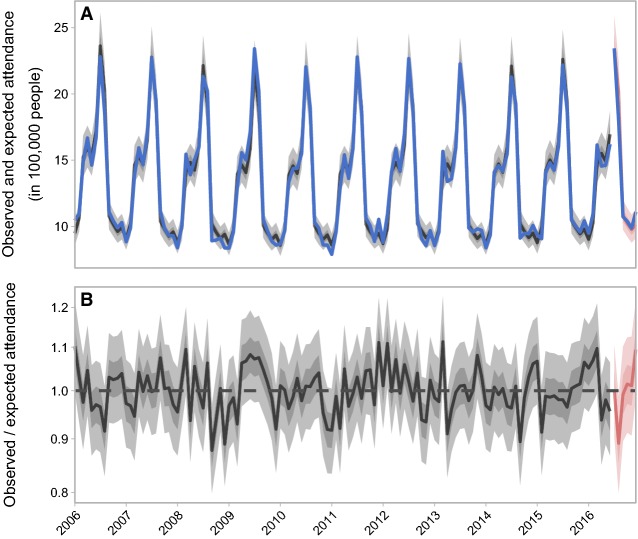
Aquarium visits: **a** number of aquarium visits (blue line) and counterfactual posterior predictive distribution based on a timeseries model (shaded band). Black band represents the time period the counterfactual model was fit to and the red band represents the forecasted posterior predictive distribution after the release of the Dory movie. **b** Ratio of observed aquarium visits (blue line in **a**) to expected aquarium visits (shaded band in **a**) where values greater than 1 represent a higher than expected number of visits. Black/red lines and dark and light shaded regions represent median, 50%, and 90% credible intervals, respectively

In the months after the movie was released, the posterior prediction from the counterfactual model suggested a low probability of an effect on aquarium visits in the USA (Fig. 4, Figs. S6, S7). For example, 1 month after *Finding Dory*, we found that the observed number of aquarium visits was close to the expected number of aquarium visits (ratio of 1.0, 90% CI 0.9–1.1). This translates to only a 0.43 probability that the visits were greater than expected. The following month, there was also a low probability that the visits were greater than expected (0.03). Four months after the movie there was still a relatively low probability of higher than expected visits (0.57).

## Discussion

Blockbuster movies can reach millions of people worldwide and play an important role in disseminating messages related to wildlife. This wide reach has often been hypothesized to give the movie industry the ability to shape consumer behaviour, potentially creating an unsustainable demand for the wildlife species they feature (Yong et al. 2011). However, this hypothesis has to date been supported by little more than anecdotal evidence (Megias et al. 2017; Silk et al. 2018). In this study, we present the first counterfactual-based impact evaluation of a blockbuster movie on demand for wildlife, using as a case-study the movie *Finding Dory*. This is also one of the first studies on the possible impact of films to use indicators of impact on biodiversity (fish import numbers) in addition to data on observed behaviour, as opposed to information on knowledge, attitudes or self-reported behavioural indicators, which can be undermined by weak links to actual behavioural outcomes (Kollmuss and Agyeman 2002; Kormos and Gifford 2014; Veríssimo et al. 2018). Thus, these results present a key opportunity to understand cause-effect relationships between human behaviour and the entertainment industry.

Our study uncovers a likely effect of the movie *Finding Dory* on online information seeking behaviour, a behaviour that according to the purchase funnel marketing model could be a precursor to purchasing, although this change was only short-term (Fig. 2). The short duration of this impact is not surprising as this type of behaviour is expected to have high plasticity (i.e. lower resistance to change) given its low effort, low emotional involvement, one-off nature and the immediacy of impact (McEachan et al. 2010). This result supports the idea that movies can move consumers through the initial stages of the purchase process (as defined by the purchase funnel model; Fig. 1). This is a finding supported by past research into movies and action to mitigate climate change where respondents were only found to be willing to change when barriers and costs were low (Balmford et al. 2004). Interestingly, our analysis also detected an even more abrupt change in search pattern in late 2014 which we believe is linked to the widely covered announcement by Pixar president Jim Morris of the first details about the movie, including the characters involved and the basic structure of the plot (Hoffman 2014; Kristobak 2014). This illustrates how these indicators can respond to a variety of events, making causal interpretation challenging without a clear counterfactual. Nonetheless, it should be noted that while the use of Google search data is increasingly seen as a way to capture a richer picture of the stages that consumers go through leading up to a potential purchase, and detecting impacts that may not be converted into actual purchases, this type of data source has limitations (Hu et al. 2014). It is important to recognize that not all searches are done in the context of a purchase and that not all consumers use Google, particularly those with more knowledge of the field who may go directly to specialist websites (Hu et al. 2014).

Despite the impacts prophesized in the popular press, we found no evidence that the movie *Finding Dory* led to an increase in actual purchases of *P. hepatus*. While this may seem surprising given the widespread coverage that the narrative received, these results highlight the differences between these two types of indicators, and how they relate to different stages in the purchasing process (Hu et al. 2014). These results align with those from past research around the *Finding Nemo* franchise and beyond. Regarding the former, Militz & Foale (2017) found no evidence of an increase in demand for clownfish or *P. hepatus* after the release of the movie *Finding Nemo.* In fact, imports of *P. hepatus* decreased substantially in the years after the movie was released. We also see no evidence of an increase in imports in the months prior to the release of the movies, as would be expected if a large spike in demand was anticipated by traders (Fig. 3). These results align with a recent analysis of the *Harry Potter* movie series, which found no evidence of an impact on the UK trade in owls or in owl abandonment after the end of the movie franchise (Megias et al. 2017).

We also did not find any evidence of *Finding Dory* driving higher visitation for US Aquaria, a behaviour hypothesized as a potential substitute for a purchase of a *P. hepatus.* This result runs contrary to qualitative research carried out in Canada, which documented several visitors stating that they decided to visit the aquarium due to movies or documentaries (Briseno-Garzon et al. 2007). Yet, given how aquarium visits are often driven by motivations linked to both children and parents and are perceived to have educational, recreational and social aims (Briseno-Garzon et al. 2007), it is possible that simple exposure to *Finding Dory* was not a strong enough incentive to have a detectable effect on visitation patterns. This is likely if we take into account the time and financial investment required for an aquarium visit. It is also possible that the movie *Finding Dory* had less impact on those most likely to visit an aquarium, which we know are more knowledgeable about marine life and conservation than the broader population of the USA (Adelman et al. 2000).

Lastly, a key challenge of the current study is that a counterfactual-based impact evaluation requires defining a counterfactual group. In this study, we opted for fish species that were similar in biological and morphological aspects to *P. hepatus,* to ensure that our analysis was not biased by other factors that may have affected public interest towards other groups of ornamental fish. This means, however, that we assumed the public is able to distinguish the species in our counterfactual group from *P. hepatus.* We believe this assumption to be true given that there are noticeable morphological differences (e.g., colour) between these species and *P. hepatus* (Fig. S1). Lastly, even if this assumption did not hold, for the counterfactual group to obscure interest in *P. hepatus*, we would have to observe an increased interest in the species in the counterfactual group. However, this scenario is not supported by the data (Figs. S2, S3).

### Evidence and credibility in conservation science

It is surprising to see conservation scientists issue warnings regarding the expected effect of *Finding Dory* on consumers (Andrews 2016; Australian Broadcasting Corporation 2016; Sohn 2016), even in the absence of credible evidence for an effect of its predecessor *Finding Nemo*. This situation suggests that assumptions around the movie’s impact on consumer demand for wildlife were deeply ingrained—even amongst scientists—which explains why references to it have also appeared in the peer-reviewed literature (Yong et al. 2011; Rhyne et al. 2012; Bush et al. 2014), despite a lack of evidence to support them.

This incongruence is important since it can affect public trust in conservation scientists as opinion leaders and in the rigour of the conservation science literature. Moreover, such claims are obviously open to rebuttal from data-driven studies, thus generating the contradictory headlines in the media that have been suggested to erode public trust in science (Resnick et al. 2015). In an era where science is seen increasingly as a partisan issue with experts being under increasing scrutiny (Gauchat 2012), it is key that scientists maintain their credibility to ensure that the historically high levels of trust in science do not decline (National Science Board 2018). One important aspect of this is ensuring a healthy level of scepticism when commenting on these emerging trends, particularly on issues where the evidence base from the peer-reviewed literature struggles to compete with the immediacy of both online and traditional media.

Beyond scientists and science, the focus on unsubstantiated threats to biodiversity is also harmful to biodiversity conservation as a global effort, as it can hijack the public’s attention from the actual processes threatening biodiversity. Given the limited attention that biodiversity conservation already receives, this seems like a misstep that conservationists cannot afford (Veríssimo et al. 2014). These inaccurate media headlines can also lead to poorly informed environmental activism and even to poorly informed environmental policy. In the case of *Finding Nemo*, restrictions on fishing for the aquarium trade were put in place in several locations in the Pacific based on public pressure stemming from the above described press accounts. This imposed a burden on local livelihoods, while delivering negligible benefits for biodiversity conservation (Militz and Foale 2017). This case offers insight into the complexity of managing ornamental fish stocks, particularly in developing countries, and highlights the importance of evidence-based natural resource management that can both benefit local livelihoods and maintain marine biodiversity.

## Conclusion

As the number of wildlife-focused movies grows, it will be increasingly important to rigorously evaluate their impact on the behaviour of their viewers and on the species they feature. Given the complexity of establishing cause-effect relationships, this effort will need to make the most of the recent advances in impact evaluation, such as the Bayesian posterior predictive counterfactual technique highlighted in this study, to build credible counterfactuals against which impact can be measured. It will also be crucial to triangulate any results using multiple datasets, particularly those that relate to behaviour of different plasticity levels. Behaviours of high plasticity (e.g. online searching) are likely to change more readily than less plastic behaviours (e.g. committing to owning an animal). Only through an evidence-based approach to understanding the effects of the media on human-wildlife relationships can conservation scientists be part of building a positive and informed relationship between society and the entertainment industry.

## Electronic supplementary material

Below is the link to the electronic supplementary material.

Supplementary material 1 (PDF 1538 kb)
