# Supplementary material for: Did the movie Finding Dory increase demand for blue tang fish?
Source: Ambio. 2019 Aug 14;49(4):903–11. doi: 10.1007/s13280-019-01233-7 (PMC7028816; doi:10.1007/s13280-019-01233-7)
Supplement: Supplementary file 1 — Supplementary material 1 (PDF 1538 kb) [file 13280_2019_1233_MOESM1_ESM.pdf]

Ambio

Electronic Supplementary Material

This supplementary material has not been peer reviewed

Title: Did the movie Finding Dory increase demand for blue tang fish?

Diogo Veríssimo[1,2,3*] , Sean Anderson[4], Michael Tlusty[5]

[1] Oxford Department of Zoology, University of Oxford, Oxford, United Kingdom

[2] Oxford Martin School, University of Oxford, Oxford, United Kingdom

[3] San Diego Zoo Institute for Conservation Research, Escondido, CA, USA

[4] Pacific Biological Station, Fisheries and Oceans Canada 3190 Hammond Bay Rd, Nanaimo, BC, V6T 6N7, Canada. Email: sean.anderson@dfo-mpo.gc.ca

[5] School for the Environment, University of Massachusetts Boston, Boston MA, USA. Email: michael.tlusty@umb.edu

*Corresponding author: Diogo Gaspar Veríssimo, Department of Zoology, University of Oxford, Oxford, OX1 3PS, UK. Email: diogo.gasparverissimo@zoo.ox.ac.uk

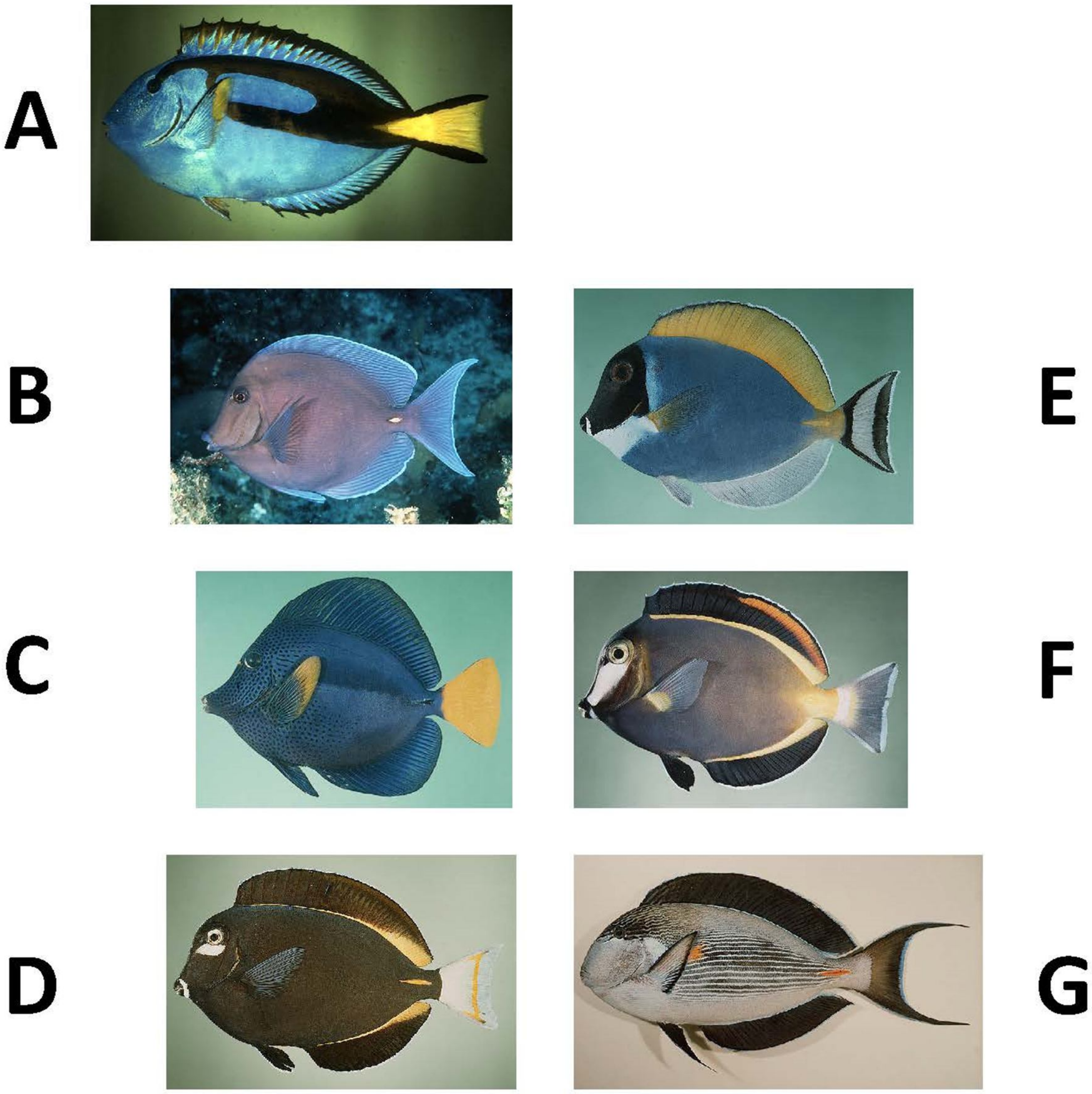

Figure S1: Photos of the species used to form the counterfactual to (A) *Paracanthurus hepatus.* (B) *Acanthurus coeruleus* (C) *Zebrasoma xanthurum* (D) *Acanthurus nigricans* (E) *Acanthurus leucosternon* (F) *Acanthurus japonicus* (G) *Acanthurus sohal*. © John E. Randall CC BY-NC 3.0

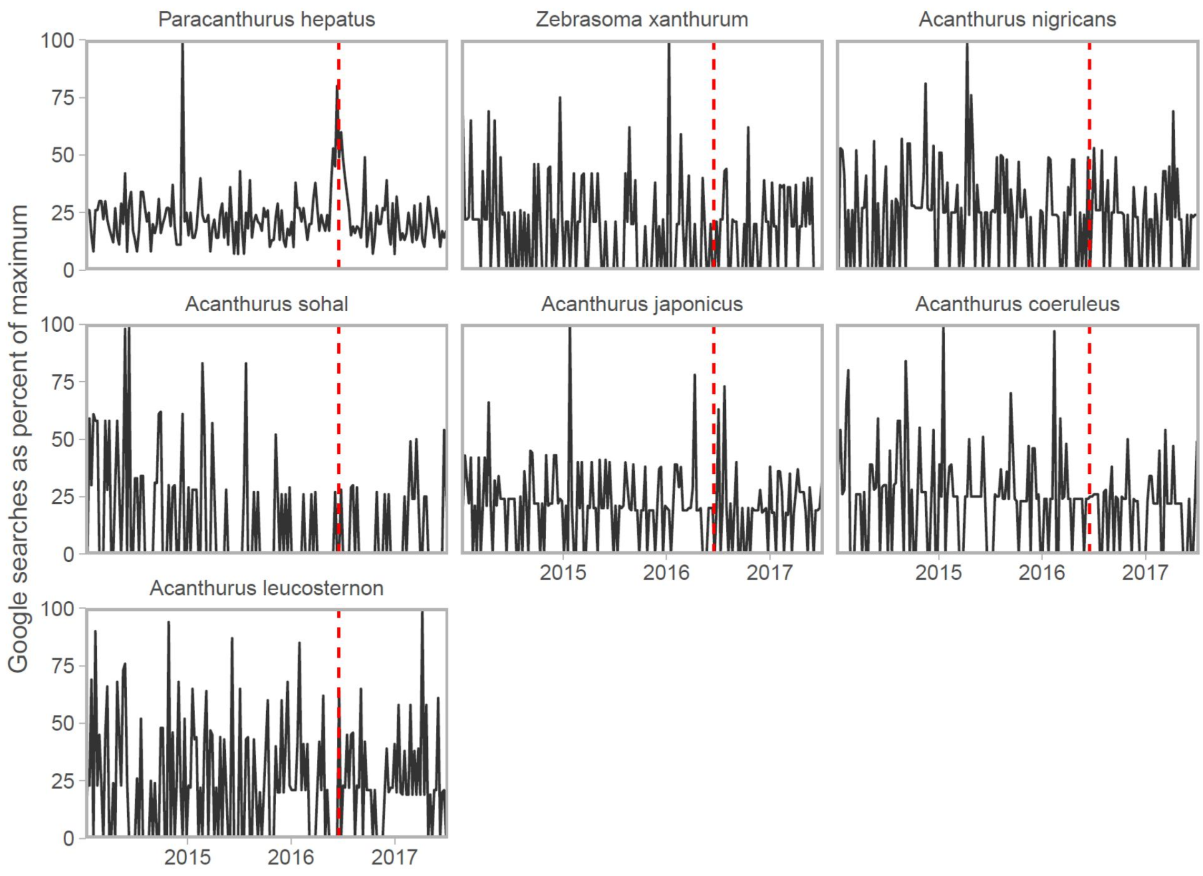

Figure S2: Google search popularity as percent of maximum during the observed time period.

The dash red line represents when the movie was released.

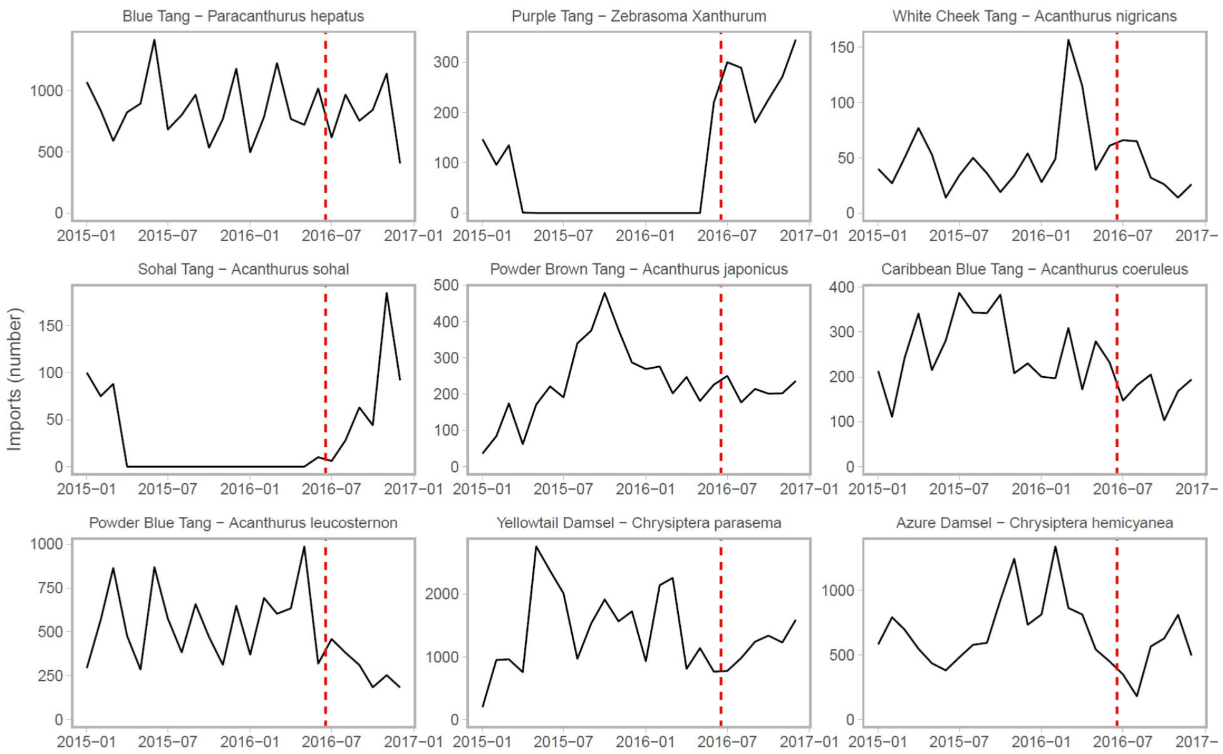

Figure S3: Imports numbers in the U.S. by species. The dashed red line represents when the movie was released.

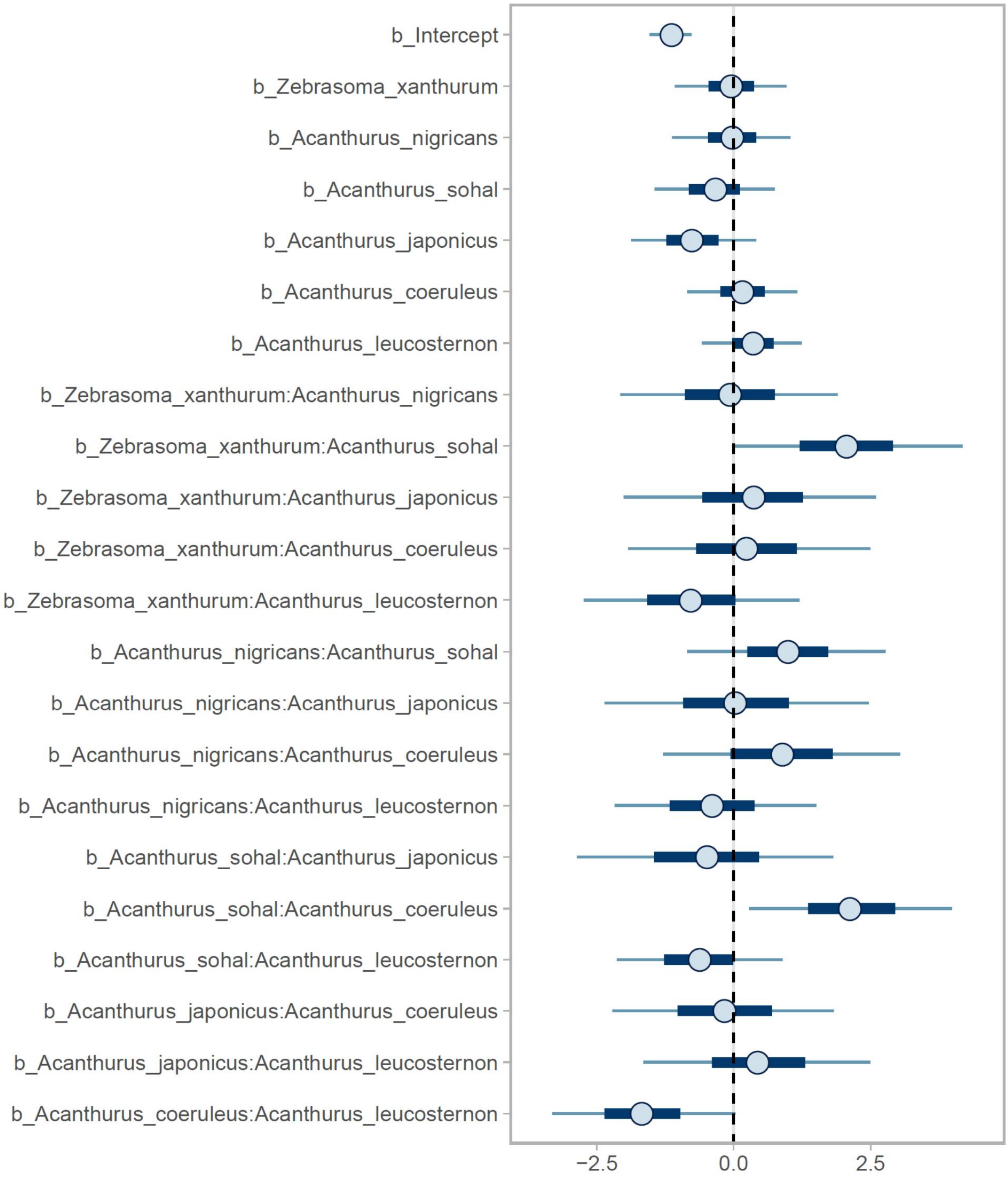

Figure S4: Coefficient estimates from the Google hits counterfactual model. Dots represent marginal posterior medians and thick and thin lines represent 50% and 90% credible intervals.

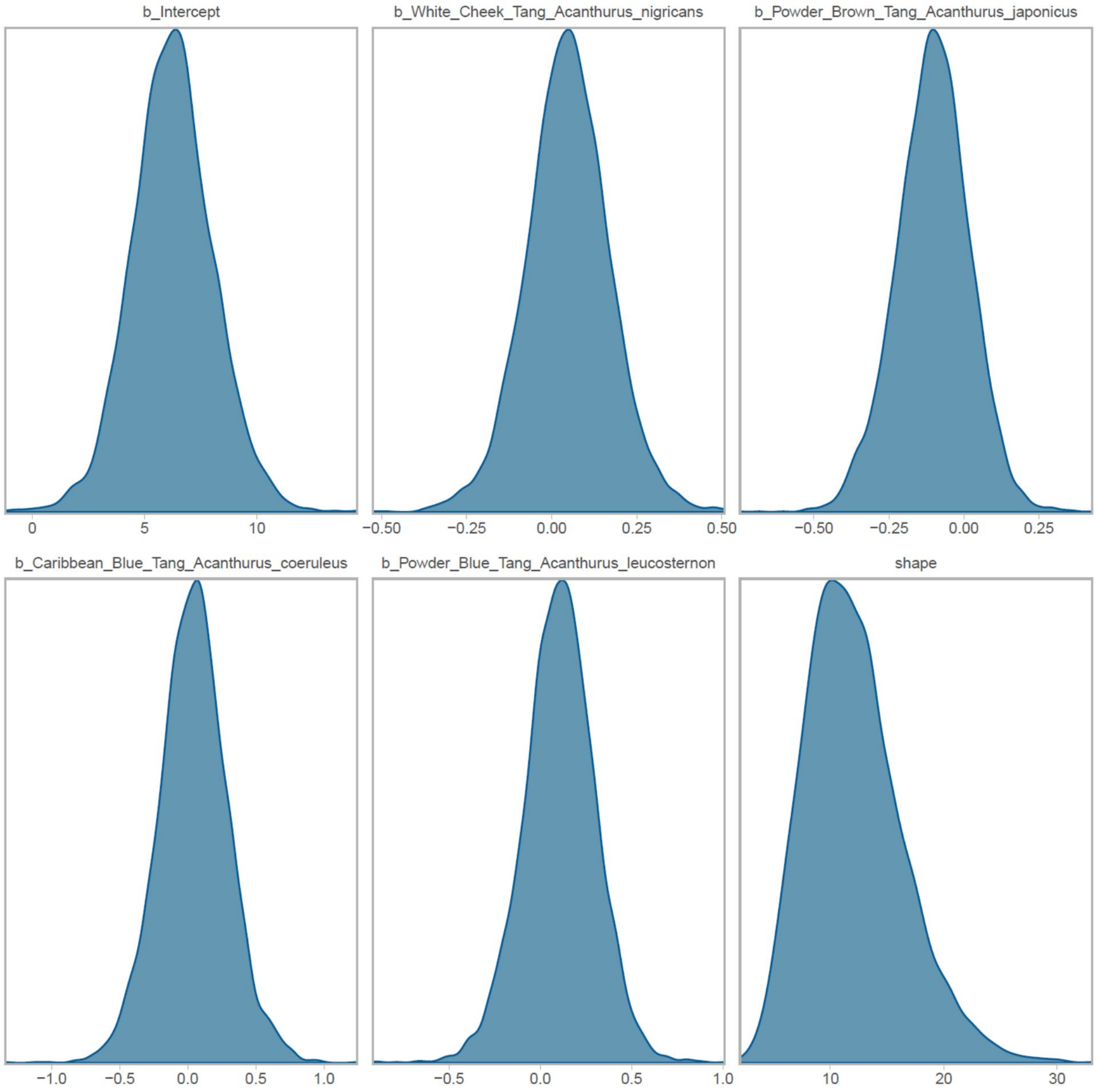

Figure S5: Coefficients for the imports counterfactual model. Shown are probability density distributions for the regression parameters. The intercept represents the estimate of average log imports of *P. hepatus* and the other β slope coefficients represent effects in log space for that particular species. The "shape" perimeter represents ϕ, the negative binomial dispersion parameter.

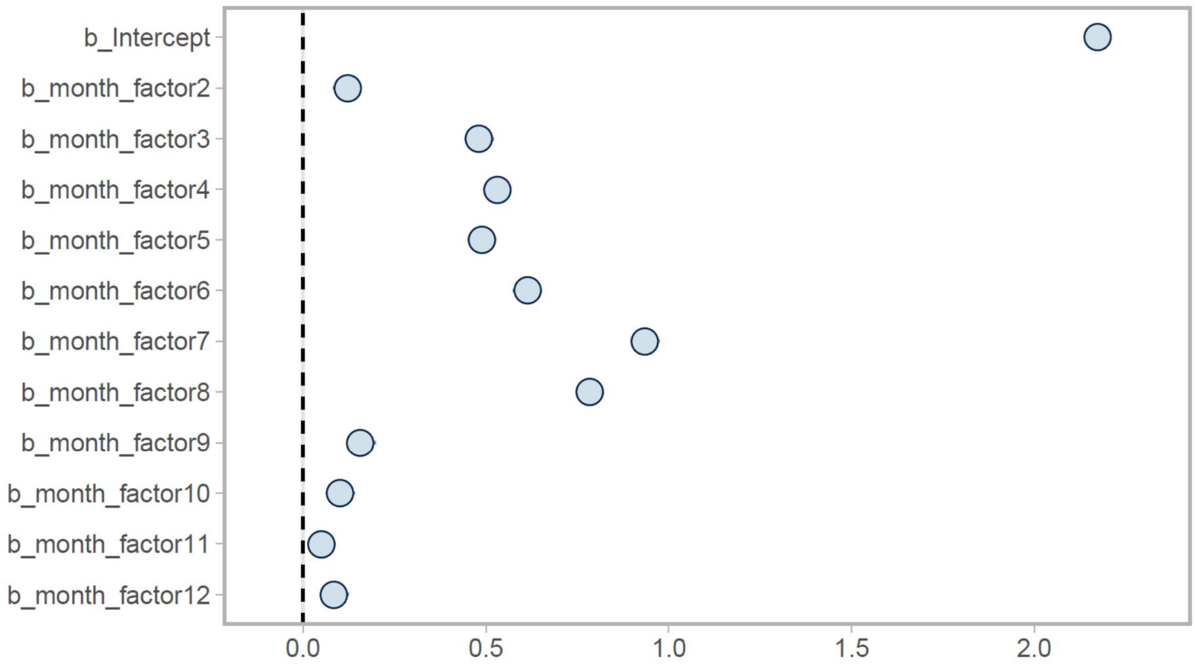

Figure S6: Coefficients for the month predictors. The intercept represents the estimate for attendance in log space (of 100,000 individuals) for January at the mean date and the other coefficients represent differences between January and that month in log space. Shown are medians (dots) and 50% and 90% credible intervals, however, the dots obscure the credible intervals here.

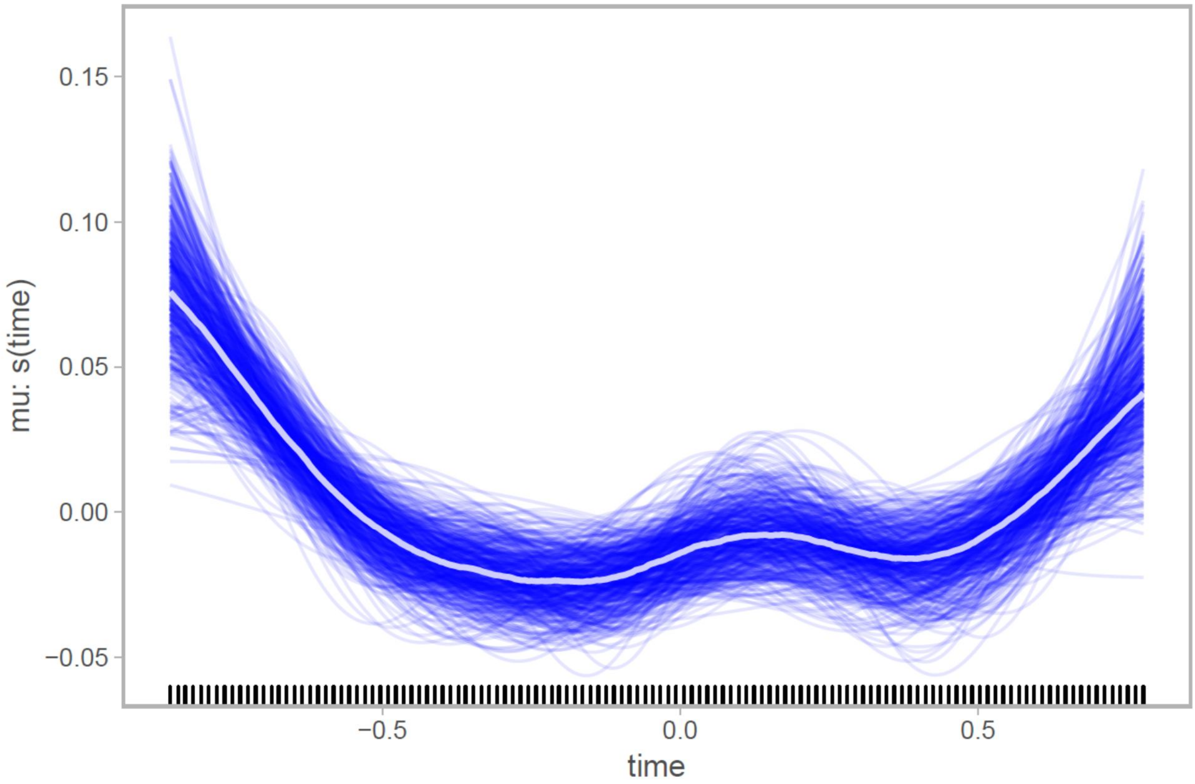

Figure S7: The Bayesian additive model spline smoother for time in the aquarium counterfactual model. The x-axis (time) has been standardized by subtracting the mean and dividing by two times the standard deviation. The white line represents the median of the posterior and the blue lines represent 500 draws from the posterior. Rug lines along the bottom represent points in time with observations.
